# Dignity Among Older Persons in Long-Term Care Institutions in Taiwan and Related Factors: A Cross-Sectional Study

**DOI:** 10.1097/jnr.0000000000000733

**Published:** 2026-03-11

**Authors:** Yu-Chi LI, Yi-Shin LIN, Shu-Ching MA, Chien-Yi WU, Hsiu-Hung WANG

**Affiliations:** 1 School of Nursing, College of Nursing, Medical University, Kaohsiung, Taiwan; 2 Department of Social Work, Meiho University, Pingtung, Taiwan; 3 Department of Senior Welfare and Services, Southern Taiwan University of Science and Technology, Tainan, Taiwan; 4 Department of Nursing, Chi-Mei Medical Centre, Tainan, Taiwan; 5 Department of Family Medicine, Kaohsiung Medical University Chung Ho Memorial Hospital, Kaohsiung, Taiwan

**Keywords:** long-term care institution, older persons, dignity, depression, activities of daily living

## Abstract

**Background:**

With a gradually aging global population, more and more older persons are receiving care in long-term care institutions during the final stages of their lives. A variety of factors, including the process of aging, dependence on assistance, and psychological problems, are known to undermine the self-perceived sense of dignity of older persons living in long-term care institutions.

**Purpose:**

The purpose of this study was to examine the factors that influence dignity and the incidence of low self-perceived dignity among older persons living in long-term care institutions.

**Methods:**

This cross-sectional descriptive study was conducted from April to December 2022 on a sample of 188 older persons living in seven long-term care institutions in southern Taiwan. A structured questionnaire, including a demographic characteristics datasheet, Barthel Index, Patient Dignity Inventory—Mandarin version, and Patient Health Questionnaire-9, was used to collect data. The independent samples *t* test, one-way analysis of variance (ANOVA), and Pearson’s correlation coefficient were used to examine the correlation between demographic variables and dignity, while binary logistic regression was used to analyze the probability of low dignity.

**Results:**

Dignity was found to relate significantly with marital status (*t* = 2.49, *p* = .014), number of children (*r* = −.19, *p* = .008), educational level (*t* = −2.42, *p* = .016), chronic condition (*r* = .25, *p* = .001), ability to perform activities of daily living (*r* = −.36, *p* < .001), dependency level (*F* = 9.85, *p* < .001), mobility level (*t* = 2.84, *p* = .005), indwelling catheter status (*t* = −3.29, *p* = .001), and depression level (*r* = .47, *p* < .001). The results of binary logistic regression analysis showed factors including having fewer children (*OR* = 0.652, *p* < .001) or no children (*OR* = 0.101, *p* = .007), a higher education level (*OR* = 3.441, *p* = .001), or lower activities of daily living (*OR* = 0.976, *p* = .001) as well as suffering from depression (*OR* = 6.025, *p* = .047) or being unable to walk (*OR* = 0.448, *p* = .045) to be significantly associated with self-perceived low dignity.

**Conclusions/Implications for Practice:**

The findings indicate that older persons living in long-term care institutions with fewer or no children, a higher level of education, and/or lower activities of daily living, as well as those unable to walk and/or suffering from depression, face a relatively greater risk of suffering from low dignity. In light of this, professional caregivers working in long-term care institutions should place greater emphasis on providing dignified care to older persons.

## Introduction

In recent decades, medical advances have contributed to the gradual increase in the average age of the global population and population aging. In 2019, the 65-and-above population numbered 703 million worldwide, with this number expected to reach 1.5 billion in 2050 (United Nations Department of Economic and Social Affairs, Population Division, 2020). Consistent with this global trend, Taiwan is confronting the severe challenges of population aging. According to statistics, the elderly population (65 and above) in Taiwan was 4.5 million (19.2%) at the end of December 2024, of whom 391,275 were using long-term care services (Directorate-General of Budget, Accounting and Statistics, Executive Yuan, Taiwan, ROC, 2025). Although life expectancy has increased, the prevalence of aging-related issues such as chronic conditions, frailty, accidental injuries, and psychological or mental distress remains high (Behzadnia et al., 2020). Currently, chronic diseases in older persons account for 23% of the global disease burden (Rababa et al., 2020). This trend leads to increased long-term care needs of older persons and to more older persons spending the rest of their lives in long-term care institutions (Rudnicka et al., 2020).

In addressing the global challenge of population aging, the United Nations established the following five principles for older persons: dignity, independence, care, participation, and self-fulfillment. These principles emphasize that, in formulating related policies, nations should regard older persons not merely as recipients of care but rather as individuals entitled to the same rights and dignity as other age groups. Furthermore, nations bear the responsibility of providing adequate support to ensure older persons can enjoy a fulfilling and dignified life in their later years (United Nations, 1991). For older persons, dignity is nearly equivalent to quality of life, with some even placing more importance on their dignity than on their lives (Kisvetrová et al., 2021).

Dignity is a common human value, and humans have the right to be respected, valued, and treated ethically. This right is regarded as an individual’s autonomous right and is also a criterion of self-worth and honor (Hofmann, 2020). Dignity is a multidimensional structure consisting of values, freedoms, meaning, hope, belonging, competency, and dignity-related perception, knowledge, and thoughts of individuals (Clancy et al., 2021). Dignity changes with time and environment and is affected by an individual’s experience, society and culture, roles and responsibilities, interpersonal interactions, and disease trajectory (Chua et al., 2022). Dignity may also be understood in terms of the relationship between health and human rights. Dignity is present in individuals with autonomy, independence, and control (Kisvetrová, Tomanová, et al., 2022). However, vulnerable and disabled patients and older persons may lose these three aspects, leading to changes in personal interaction experiences (Banerjee et al., 2021; Clancy et al., 2021). Given the asymmetrical relationship between health and human rights, the dignity of these older individuals may be threatened if medical professionals do not provide care and attention (Kerr et al., 2020; Lindwall & Lohne, 2021). Older persons residing in long-term care institutions are at particular risk of losing their dignity (Albers et al., 2013). Compared to community-dwelling elderly individuals, those in long-term care institutions not only face threats to their dignity due to functional and/or cognitive decline but also experience unfamiliar living environments, limited privacy, heavy reliance on staff, and a progressively shrinking social network, all of which make them highly vulnerable to dignity loss (Albers et al., 2013; van Dammen et al., 2025). Professional caregivers working in long-term care institutions should be aware of the importance of dignity and preserve the dignity of older persons during their work.

Previous studies have shown that dignity in older persons relates to physical impairment, increased dependency, lack of personal privacy, lack of social contact, lack of autonomy, and lack of independence (Chua et al., 2022; Kerr et al., 2020). A study on older people living in long-term care institutions in Czechia found that physical function and dependency severely affect dignity perceptions. Therefore, not being a burden on others is extremely important to older persons (Kisvetrová, Tomanová, et al., 2022). According to a Swedish study examining dignity in older adult residents of long-term care institutions, disability, increased dependency, and worries about the future all influence the dignity perceptions of older people living in long-term care institutions (Söderman et al., 2023). In addition, psychological problems, depression in particular, may affect the dignity perceptions of older adults (Salehi et al., 2020). Therefore, dignity preservation and depression evaluation are considered to be important issues in elder care (Kisvetrová, Tomanová, et al., 2022). Other factors that affect dignity in older persons include gender, age, living with a partner, and religious beliefs, all of which have a role in shaping the life-value system of older individuals (Albers et al., 2013).

All of the research on dignity in older people living in long-term care institutions cited above was conducted overseas (Kisvetrová, Tomanová, et al., 2022). Similar research has been rare in Taiwan. Therefore, the purpose of this study was to examine the dignity perceived by older persons living in long-term care institutions in Taiwan, the related factors, and the predictors and incidence of low dignity to provide a reference for professional caregivers working in these institutions.

## Methods

### Study Design, Setting, and Participants

This cross-sectional descriptive correlation study employed convenience sampling. Enrollment was carried out in seven long-term care institutions in southern Taiwan from April to December 2022. Each of these seven long-term care institutions was licensed and maintained with between 40 and 75 beds. All of the older persons enrolled had experience with acute disease, chronic disease progression, or injury, and with decreased physical function or mobility. Inclusion criteria were (1) living in one of the targeted long-term care institutions; (2) aged 65 years or above; (3) able to communicate in Mandarin and Taiwanese Hokkien; and (4) willing to voluntarily participate after understanding the purpose and process of this study and signing informed consent. Otherwise qualified individuals who either (1) had a diagnosis of dementia, delusion, or other organic brain disease or (2) had impaired consciousness or incapacity were excluded.

### Instruments

To investigate factors related to dignity in older persons living in long-term care institutions, a structured questionnaire was used for data collection. The instruments included in this questionnaire were a demographic characteristics datasheet, the Barthel Index (BI), Patient Dignity Inventory—Mandarin Version (PDI-MV), and Patient Health Questionnaire-9 (PHQ-9).

#### Demographic characteristics

The demographic characteristics data collected included age, gender, marital status, number of children, educational level, chronic condition, dependency level, mobility level, and indwelling catheter status. A chronic condition was defined as an illness requiring ongoing medical care or that limits daily activities, such as heart disease, hypertension, diabetes, chronic obstructive pulmonary disease, cancer, kidney disease, and arthritis (Watson et al., 2024). Mobility level refers to the ability of an individual to move, whether independently, with assistive devices, or with help from others (Villalobos Dintrans, 2025). This study categorized mobility level into four groups: normal walking, walking with a walker, using a wheelchair, and bedridden. Furthermore, older persons able to walk independently or with a walker were classified as “able to walk,” while those who needed to use a wheelchair or were bedridden were classified as “unable to walk” (Villalobos Dintrans, 2025).

#### Barthel Index

BI, widely used to evaluate activities of daily living (ADL), is primarily designed to measure degeneration status and treatment effects in patients undergoing rehabilitation and older patients (Ocagli et al., 2021). BI includes 10 evaluation items: feeding, transfers, grooming, toilet use, bathing, mobility on level surfaces, stairs, dressing, bowel control, and bladder control (Ocagli et al., 2021). The total score ranges from 0 to 100 points, with lower scores indicating less sufficient self-care ability and higher dependency. The five dependency grades include total dependence (0–20 points), severe dependence (21–60 points), moderate dependence (61–90 points), mild dependence (91–99 points), and total independence (100 points; Pashmdarfard & Azad, 2020). The Cronbach’s α of BI was previously found to be .94 to .96 (Ocagli et al., 2021; Pashmdarfard & Azad, 2020), and was .91 in this study.

#### Patient Dignity Inventory—Mandarin Version

Developed by Canadian researchers Chochinov et al. (2008), the English version of the PDI is used to measure self-perceived dignity over the previous few days. The Patient Dignity Inventory—Mandarin Version (PDI-MV) was translated by Li et al. (2018) in Taiwan. The PDI-MV includes the four dimensions of existential distress, loss of support and meaning, symptom distress, and loss of autonomy. Each of the 25 questions on the inventory is scored on a 5-point Likert scale (1 = *not a problem*, 2 = *a slight problem*, 3 = *a problem*, 4 = *a major problem*, 5 = *an overwhelming problem*), with a total possible score range of 25–125 and higher scores indicating lower dignity (Li et al., 2018). A total PDI-MV score ≥35 indicates low dignity, with significant sensitivity and specificity for psychological problems. For example, the sensitivity and specificity for demoralization are 84.9% and 79.1%, respectively, and the sensitivity and specificity for depression are 73.8% and 70.9%, respectively (Li et al., 2023). The participants in this study were divided based on PDI-MV score into two groups: low dignity (PDI-MV ≥35) and non-low dignity (PDI-MV ≤35). The Cronbach’s α values for the total scale and its four dimensions were .95 and .83–.95, respectively, in Li et al. (2018), and the Cronbach’s α was .84 in this study.

#### Patient Health Questionnaire-9

The original English version of the PHQ-9, developed by the U.S. researchers Kroenke et al. (2010), is widely used to measure the degree of patient depression over the previous 2-week period. The Mandarin version of the PHQ-9 was translated by Taiwanese researchers Liu et al. (2011). The PHQ-9 contains nine questions, all scored on a 4-point Likert scale (from 0 = *not at all* to 3 = *almost every day*), with higher scores indicating a greater likelihood of depression. PHQ-9 ≥10 is indicative of depression (Liu et al., 2011). The Cronbach’s α of PHQ-9 was .80–. 89 in prior studies (Liu et al., 2011; Sun et al., 2020) and was .80 in this study.

### Sample Size

G*Power 3.1.9.7 was used to calculate the minimum sample size. Due to the nature of the outcome variable (consisting of two groups, with total PDI-MV ≥35 indicating low dignity and ≤35 indicating non-low dignity; Li et al., 2023), binary logistic regression was used to analyze the results. A previous study was reviewed, and two-tailed, α = .05, power = 0.9, odds ratio =1.78 (Fuseini, Rawson, Redley, et al., 2023), *P* (*Y*=1) = 0.30 (Fuseini, Rawson, Redley, et al., 2023), *X* distribution = normal, and default system values for *X* mean and *SD* were used. The minimum required sample size was 167.

### Data Collection Procedures

One investigator with a PhD degree, more than 20 years of experience in long-term care, and the ability to speak Mandarin and Taiwanese Hokkien was employed to collect the study data. Before beginning the study, the principal investigator explained the content of the study instruments and enrollment procedures to the investigators. Over the study period, the investigators periodically enrolled eligible participants from long-term care institutions and performed questionnaire surveys. Before carrying out the questionnaire survey, the investigators explained to potential participants the study purposes and procedures and that participants had the right to refuse to complete the questionnaire. Participants filled out the questionnaire survey only after they had signed informed consent. The questionnaire was completed by the participant either on their own or through a face-to-face interview with the investigator. The investigator guided the participant during the questionnaire completion process. If the participant had any questions, the investigator would explain or clarify on the spot.

### Ethical Considerations

Data collection in this study complied with ethical principles. Approval was granted by the institutional review board (no. 11101-010) before implementation. The investigator explained the study purposes and procedures to the participant, obtained their consent, and made sure they completed the informed consent form before starting the questionnaire survey. This study collected data in a confidential and anonymous manner, and participant identification data was coded to protect privacy. All participants had the right to withdraw from the study at any time. If a participant had an emotional management need, the investigator would provide professional assistance at appropriate times. The results of the data analysis were used for academic research purposes only.

### Statistical Analysis

After coding the study data, IBM SPSS 26.0 (IBM Corp., Armonk, NY, USA) was used for data analysis. Frequency, percentage, mean, and standard deviation were used for descriptive statistical analysis; independent samples *t* test, one-way ANOVA, and Pearson’s correlation coefficient were used to examine the correlation between demographic variables and dignity; and binary logistic regression was used for inferential statistical analysis.

The study data were independent (Durbin–Watson = 1.698 > dU = 1.637) and normally distributed (Kolmogorov–Smirnov = 0.200, *p* = .091). The scatter plot of standardized residue and predicted value found that the data fluctuated around the 0 line and met the hypothesis of homogeneous variance. Data variance homogeneity tests for two groups and for three or more groups (Levene’s test) were not significant (*p* > .05). The ANOVA regression model was suitable (*p* < .001). The above results demonstrate that the study data were suitable for prediction and explanation. For all statistical analysis results, *p* < .05 was used to indicate statistical significance.

## Results

### Participant Demographic Characteristics

One hundred ninety-five questionnaires were distributed, with 188 valid responses received (valid response rate: 96.4%). The participants included 106 men (56.4%) and 82 women (43.6%), with ages ranging from 65 to 95 years (*M* ± *SD* = 76.90 ± 7.86). Most of the participants were married (84.6%), had children (77.7%), had an elementary school education only (39.3%), and had a chronic condition history (87.2%). Also, most were wheelchair-bound (61.2%) and did not have an indwelling catheter (80.9%). Slightly less than half were severely dependent (48.4%) and had low dignity (48.9%; PDI-MV ≥35). Only 5.3% suffered from depression (PHQ-9 ≥10; Table 1).

**Table 1:** Participant Demographic Characteristics (*N*=188)

Variable	*n* (%)
Age (years, mean and *SD*; range 65–95)	76.90 (7.86)
Gender	
Male	106 (56.4)
Female	82 (43.6)
Marital status	
Single	29 (15.4)
Married	159 (84.6)
Children (Mean and *SD*, range 0–7)	2.29 (1.61)
No	42 (22.3)
Yes	146 (77.7)
Educational level	
No	40 (21.3)
Elementary school	74 (39.3)
Junior high school	47 (25.0)
Senior high school	27 (14.4)
Chronic condition (Mean and *SD*, range 0–7)	1.83 (1.25)
No	24 (12.8)
1	58 (30.8)
2	55 (29.3)
≥3–7	51 (27.1)
Barthel Index (Mean and *SD*, range 0–95)	47.18 (27.93)
Scoring 91–99 (mild dependence)	14 (7.5)
Scoring 61–90 (moderate dependence)	38 (20.2)
Scoring 21–60 (severe dependence)	91 (48.4)
Scoring 0–20 (complete dependence)	45 (23.9)
Mobility level	
Normal	34 (18.1)
Walker	30 (15.9)
Wheelchair	115 (61.2)
Bedridden	9 (4.8)
Indwelling catheter status	
No	152 (80.9)
Foley tube	30 (15.9)
Nasogastric tube	6 (3.2)
PDI-MV (Mean and *SD*; range 25–76)	37.21 (8.75)
Scoring <35 (no low dignity)	96 (52.1)
Scoring ≥35 (low dignity)	92 (48.9)
PHQ-9 (Mean and *SD*, range 0–20)	3.86 (3.06)
Scoring <10 (no depression)	178 (94.7)
Scoring ≥10 (depression)	10 (5.3)

*Note.* PDI-MV = Patient Dignity Inventory—Mandarin Version; PHQ-9 = Patient Health Questionnaire-9.

### Dimension Mean Scores of the Patient Dignity Inventory—Mandarin Version

PDI-MV scores in this study ranged from 25 to 76 (*M* ± *SD* = 37.21 ± 8.75), with higher PDI-MV scores indicating lower dignity. The mean scores of the four dimensions of PDI-MV were existential distress (total *M* ± *SD* = 20.82 ± 5.20, item *M* ± *SD* = 1.39 ± 0.35), loss of support and meaning (total *M* ± *SD* = 5.02 ± 1.45, item *M* ± *SD* = 1.26 ± 0.36), symptom distress (total *M* ± *SD* = 6.06 ± 2.02, item *M* ± *SD* = 1.51 ± 0.51), and loss of autonomy (total *M* ± *SD* = 5.31 ± 3.19, item *M* ± *SD* = 2.65 ± 1.60; Table 2). Among these dimensions, the highest item mean scores were for loss of autonomy and symptom distress.

**Table 2:** Patient Dignity Inventory—Mandarin Version: Dimension Mean Scores (*N* = 188)

Dimension	Total *M* ± *SD*	Items	Item *M* ± *SD*	Rank
Existential distress	20.82±5.20	15	1.39±0.35	3
Loss of support and sense of meaning	5.02±1.45	4	1.26±0.36	4
Symptom distress	6.06±2.02	4	1.51±0.51	2
Loss of autonomy	5.31±3.19	2	2.65±1.60	1

*Note.* Total *M* = sum of scores for that dimension divided by total number of participants (*N* = 188); Item *M* = sum of scores for that dimension divided by total number of participants (*N* = 188) and the number of items.

### Correlations and Mean Difference Between Demographic Characteristics and Dignity

Significant correlations were identified between certain demographic characteristics and dignity, as measured by the PDI-MV score. Specifically, the number of children (*r* = −.19, *p* = .008) and BI score (*r* = −.36, *p* < .001) were significantly negatively correlated with PDI-MV score. Having fewer children or a lower BI score (indicating greater dependency) was associated with higher PDI-MV scores, reflecting lower dignity. In addition, the number of chronic conditions (*r* = .25, *p* = .001) and PHQ-9 score (*r* = .47, *p* < .001) correlated significantly and positively with PDI-MV score, suggesting participants with more chronic conditions or a higher PHQ-9 score (indicating more severe depression) also had higher PDI-MV scores, reflecting lower dignity.

Furthermore, significant differences in dignity were observed based on several demographic characteristics, specifically in single versus married participants (*t* = 2.49, *p* = .014), those with and without children (*t* = 3.07, *p* = .002), (*t* = −2.42, *p* = .016), those with two or more versus one chronic condition (*t* = −2.28, *p* = .024), those with complete versus mild to severe dependence (*F* = 9.85, *p* < .001), those unable versus able to walk (*t* = 2.84, *p* = .005), those with versus without an indwelling catheter (*t* = −3.29, *p* = .001), and those with versus without depression (*t* = −5.46, *p* = .021; Table 3).

**Table 3:** Correlations and Mean Difference Between Demographic Characteristics and Dignity (*N* = 188)

Variable	*n* (%)	*M* ± *SD*	*r* /*t*/*F*	*p*	Post Hoc ^d^
Age (years)			0.11 ^a^	.138	
Gender			0.58 ^b^	.954	
Male	106 (56.4)	37.25±8.54			
Female	82 (43.6)	37.17±9.08			
Marital status			2.49 ^b^	.014	
Single	28 (14.9)	40.96±9.32			
Married	160 (85.1)	36.56±8.51			
Children			−0.19 ^a^	.008	
No	42 (23.3)	40.79±8.32	3.07 ^b^	.002	
Yes	146 (77.7)	36.18±8.63			
Educational level			−2.42 ^b^	.016	
Elementary school or below	114 (60.6)	35.98±8.85			
Junior high school or above	74 (39.4)	39.11±8.32			
Chronic condition			0.25 ^a^	.001	
≤1	80 (42.6)	35.54±6.67	−2.28 ^b^	.024	
≥2–7	108 (57.4)	38.45±9.87			
Barthel Index			−0.36 ^a^	<.001	
① Mild dependence	14 (7.5)	33.36±8.15	9.85 ^c^	<.001	④ > ①
② Moderate dependence	38 (20.2)	33.92±8.49			④ > ②
③ Severe dependence	91 (48.4)	36.51±7.89			④ > ③
④ Complete dependence	45 (23.9)	42.62±8.54			
Mobility level			2.84 ^b^	.005	
Unable to walk	124 (66.0)	38.49±8.82			
Able to walk	64 (35.0)	34.73±8.13			
Indwelling catheter status			−3.29 ^b^	.001	
No	152 (80.9)	36.22±8.26			
Yes	36 (19.1)	41.42±9.63			
Depression status (PHQ-9 ≥10)			0.47 ^a^	<.001	
No	178 (94.7)	36.44±7.49	−5.46 ^b^	.021	
Yes	10 (5.3)	50.90±16.34			

*Note.* Means and *SDs* refer to scores on the Patient Dignity Inventory—Mandarin Version. PDI-MV = Patient Dignity Inventory—Mandarin Version; PHQ-9 = Patient Health Questionnaire-9.

^a^ Pearson’s correlation. ^b^ *t* text. ^c^ *F* test. ^d^ Scheffe’s method.

### Binary Logistic Regression for the Variables Related to Low Dignity

Binary logistic regression was used in this study to construct a regression model to predict the probability of developing low dignity. The participants were divided for this purpose into low dignity (PDI-MV ≥35) and non-low dignity (PDI-MV ≤35) groups (Li et al., 2023).

Binary logistic regression analysis results showed that each decrease in the number of children (*OR* = 0.652, *p* < .001), each one-unit decrease in BI score (*OR* = 0.976, *p* = .001), and each increase in PHQ-9 score (*OR* = 1.309, *p* < .001) increases the probability of having low dignity by 65.2%, 97.6%, and 130.9%, respectively. Being without children (vs. having children; *OR* = 0.101, *p* = .007), having a junior high school or above level of education (vs. having an elementary school or below level; *OR* = 3.441, *p* = .001), being unable to walk (vs. being able to walk; *OR* = 0.448, *p* = .045), and having depression (vs. not having depression; *OR* = 6.025, *p* = .047) were found to increase the probability of having low dignity by 10.1%, 271.3%, 43.7%, and 602.5%, respectively (Table 4).

**Table 4:** Binary Logistic Regression for the Variables Related to Low Dignity

Variable	*B*	*SE*	*p*	Exp (*B*)	95% CI
Gender (Ref: Female)	0.139	0.336	.679	1.149	[0.59, 2.22]
Age (years)	0.008	0.024	.746	1.008	[0.96, 1.05]
Number of children	−0.428	0.123	<.001	0.652	[0.51, 0.83]
Number of chronic conditions	0.205	0.148	.168	1.227	[0.92, 1.64]
Score of the Barthel Index	−0.024	0.007	.001	0.976	[0.96, 0.99]
Score of PHQ-9	0.269	0.070	<.001	1.309	[1.14, 1.50]
Marital status (Ref: Married)	1.121	0.931	.229	3.068	[0.49, 19.04]
Children (Ref: Yes)	−2.291	0.843	.007	0.101	[0.02, 0.53]
Education level (Ref: Elementary school or below)	1.236	0.360	.001	3.441	[1.69, 6.97]
Chronic condition (Ref: No)	0.635	0.357	.075	1.887	[0.94, 3.79]
Indwelling catheter (Ref: No)	0.390	0.455	.391	1.478	[0.61, 3.61]
Mobility level (Ref: able to walk)	−0.802	0.401	.045	0.448	[0.20, 0.98]
Depression (Ref: No)	1.796	0.904	.047	6.025	[1.03, 35.44]

*Note.* CI = confidence interval; Ref = reference; PHQ-9 = Patient Health Questionnaire-9.

Binary logistic regression analysis results showed that those participants with fewer children, without any children, a junior high school or higher level of education, or a low BI score, and those unable to walk, earning a higher PHQ-9 score, or affected by depression were more likely to have low dignity.

## Discussion

The purpose of this study was to examine self-perceived dignity in older persons living in long-term care institutions and its related factors, as well as to estimate the risk of persons in this population developing low dignity. The participants in this study had a mean dignity score of 37.21 (*SD* = 8.75), indicating a slight problem with dignity. These findings align with previous studies. For example, a study conducted in the Czech Republic used the PDI to measure the dignity of 470 home-based and hospitalized older persons, reporting that mean dignity scores ranged from 43.9 (*SD* = 16.90) to 52.8 (*SD* = 16.0), indicating a slight-to-moderate problem with dignity (Kisvetrová, Mandysová, et al., 2022). Similarly, a study conducted in Iran found mean dignity scores among community-dwelling older persons to range from 36.91 (*SD* = 12.44) to 57.38 (*SD* = 19.03), also indicating a slight-to-moderate problem with dignity (Moradoghli et al., 2022). Also, a study in Ghana that used the Hospitalized Older Adults’ Dignity Scale reported a mean dignity score of 44.9 (*SD* = 15.6) among 270 hospitalized older adult participants, indicating low-to-moderate dignity problems (Fuseini, Rawson, Redley, et al., 2023). Despite differences in geographic regions, older populations, and measurement tools, these studies consistently highlight that the dignity issues faced by older persons are widespread, emphasizing the importance of assessing and maintaining self-perceived dignity in older individuals.

In terms of PDI-MV dimensions, mean scores for loss of autonomy and symptom distress were higher than those of other dimensions. This finding is consistent with previous studies that have identified strong links between autonomy and independence in daily activities and subjective feelings of well-being and dignity in older persons (Albers et al., 2013; Kisvetrová, Tomanová, et al., 2022). Aging, poor health, and physical discomfort restrict the ADL abilities of older persons, increasing their dependence on others and leading to a loss of dignity (Kisvetrová, Tomanová, et al., 2022; Schreier et al., 2016). Therefore, it is crucial to enable older persons living in long-term care institutions to maintain autonomy and independence as much as their health allows.

In terms of demographic variables, single older persons were found to have significantly lower self-perceived levels of dignity than their married counterparts, which is consistent with previous findings (Karimi et al., 2019; Moradoghli et al., 2022). However, participants with higher educational levels were also shown to exhibit significantly lower levels of dignity, which contradicts previous findings (Martín-Abreu et al., 2022). In addition, in another previous study, no significant association was reported between dignity and either marital status or educational level (Fuseini, Rawson, Redley, et al., 2023). These discrepancies in study results indicate that the relationship between marital status, education level, and dignity requires further exploration. In this study, the number of children was also identified as a factor of influence on dignity. Although this association has not been widely investigated in the literature, the concept of identity dignity suggests that social roles (e.g., being a parent, spouse, or family member) impact dignity in individuals profoundly (Pageau et al., 2024). Identity dignity is shaped by social interactions and may be affected by aging, illness, or hospitalization (Kane & de Vries, 2017; Pageau et al., 2024). Therefore, this may provide an explanation for the lower dignity observed among older adult residents of long-term care institutions with fewer or no children.

The findings of this study also found a significant association between dignity and the number of chronic conditions, ADL, dependency, mobility status, and having an indwelling catheter. Consistent with previous studies, having a frail constitution, more chronic conditions, physical impairment, decreased ADL, and high dependency were all shown to be associated with lower dignity (Albers et al., 2013; Moradoghli et al., 2022; Schreier et al., 2016). These factors, which directly impact the autonomy, independence, and control of individuals, are the greatest threats to and determinants of dignity (Kerr et al., 2020; Lindwall & Lohne, 2021). Therefore, professional caregivers should develop chronic condition prevention and management plans to improve the physical fitness and daily functioning of older persons. Also, providing leisure, physical, and rehabilitation activities as well as opportunities for choice and participation can enhance the sense of control that older persons perceive over their lives, thereby bolstering their dignity (Lin & Yen, 2018; Schreier et al., 2016).

The findings of this study also revealed depression as a predictor of loss of dignity in this population (Kisvetrová, Tomanová, et al., 2022). Dignity includes self-esteem, defined as the dignity that individuals attribute to themselves (Kane & de Vries, 2017). However, people with depression are often unable to view themselves positively, often perceiving lower dignity during periods of depression (Jacobson, 2009). Therefore, professional caregivers should not only understand the psychological state of older persons but also respect their dignity. Dignified care that protects their privacy, provides options and opportunities to participate in decision-making, is communicated effectively, and offers empathetic care supportive of their role, contribution, and value should be provided to older persons to alleviate their depression and improve their dignity (Fuseini, Rawson, Ley, et al., 2023; Salehi et al., 2020).

The significant contributions of this study center primarily on providing important insights into predicting the risk of low dignity in institutionalized older adults and identifying the predictors of low dignity in this population. The issues addressed in this study have rarely been addressed in other studies in the literature. Based on the PDI total score classification criteria used (Chochinov et al., 2008), scores of 26–50 indicate a mild dignity problem, while scores above 50 indicate a moderate-to-severe dignity problem. Few studies have investigated the appropriate cutoff values for PDI total scores. Li et al. (2023) proposed a total PDI score ≥35 as the criterion for low dignity, demonstrating significant sensitivity and specificity in detecting psychological issues such as depression (Li et al., 2023). Based on this criterion, this study categorized participants into either the low dignity (PDI-MV ≥ 35) or non-low dignity (PDI-MV ≤ 35) group, and conducted binary logistic regression analysis to predict the respective factors and probabilities of having low dignity in both. Based on the findings, having fewer or no children, a lower BI score (lower ADL), a higher PHQ-9 score (depression), or a higher education level, as well as being unable to walk, all significantly increased the risk of low dignity, with the odds ranging from 10.1% to 602.5%. These findings provide a reference for long-term care professionals working to formulate dignified care strategies.

Given current population aging trends, professional caregivers working in long-term care institutions must understand the factors affecting dignity and be equipped with the knowledge and skills to provide dignified care. In working to foster person-centered long-term care, efforts should be made to improve the related knowledge and skills taught in geriatric physiology, psychology, exercise, rehabilitation, and recreation education programs, as well as involve older persons in care planning to ensure their individual needs and preferences are respected (Banerjee et al., 2021; Kim et al., 2024; Kisvetrová, Tomanová, et al., 2022).

### Limitations

This study exclusively recruited older persons receiving full-day care in seven long-term care institutions in southern Taiwan. Thus, the findings may not be generalizable to older populations in other regions or to those receiving care in a different format, for example, home- or community-based services. In addition, as the study was carried out during the post–COVID-19 pandemic, the potential influence of postpandemic changes to institutional policies, health care services, and social interactions on the dignity perceptions of older persons should be investigated further. Moreover, this study used a cross-sectional design, and data were collected at a single point in time. Thus, long-term changes in dignity could not be tracked in the participants. Based on these limitations, caution should be exercised in generalizing the findings to other populations or settings.

### Conclusions

Based on the findings of this study, the self-perceived dignity of older persons living in long-term care institutions correlates significantly with the following variables: marital status, number of children, educational level, chronic condition status, ADL status, dependency level, mobility, indwelling catheter status, and depression. Thus, older individuals residing in long-term care institutions who have few or no children, a higher education level, lower ADL, and/or higher dependency, who are unable to walk, or are affected by depression, may be at higher risk of developing low dignity. Hence, professional caregivers should pay more attention to the issue of dignity in older persons living in long-term care institutions. Being able to rapidly and accurately evaluate, identify, and resolve dignity issues among older adult residents of long-term care institutions is an increasingly indispensable skill for professional caregivers. In light of the findings of this study, professional caregivers should monitor the dignity level of older adults living in long-term care institutions routinely and provide dignified care to preserve and enhance their dignity. To more fully clarify and understand the dignity perceptions of older populations, future research should be expanded to include community-dwelling older persons.
